# Controversies in dengue pathogenesis

**DOI:** 10.1179/2046904712Z.00000000045

**Published:** 2012-05

**Authors:** Scott B Halstead

**Affiliations:** Dengue Vaccine Initiative, International Vaccine Institute, Seoul, Korea

**Keywords:** Dengue, Classification, Case definition, Virus, T cell, Auto-immune

## Abstract

Research into the pathogenesis of dengue fever has exploded over the last half-century, with issues that were considered simple becoming more complex as additional data are found. This has led to the development of a number of controversies that are being studied across the globe and debated in the literature. In this paper, the following six controversies are analysed and, where possible, resolved: the 1997 World Health Organization (WHO) case definition of dengue haemorrhagic fever (DHF) is not useful; DHF is not significantly associated with secondary dengue infection; DHF results from infection with a ‘virulent’ dengue virus; DHF is owing to abnormal T-cell responses; DHF results from auto-immune responses; and DHF results from direct infection of endothelial cells.

## Background

The world is in the midst of a dengue pandemic and more than 1000 papers are added each year to the literature on dengue. Clinicians and scientists attempting to understand the pathogenesis of severe dengue are confronted by six major controversies: (i) the 1997 World Health Organization (WHO) case definition of dengue haemorrhagic fever (DHF) is not useful; (ii) DHF is not significantly associated with secondary dengue infection; (iii) DHF results from infection with a ‘virulent’ dengue virus; (iv) DHF is caused by abnormal T-cell responses; (v) DHF results from auto-immune responses; and (vi) DHF results from direct infection of endothelial cells.

Each will be considered briefly in this article.

### (i) The 1997 WHO Case Definition is Not UsefulWhat is the 1997 WHO case definition of DHF/DSS?

Clinical application of the 1997 WHO case definition of DHF/dengue shock syndrome (DSS) (Box 1) had several problems. Firstly, the tourniquet test and thrombocytopenia have low positive predictive values,^1^–^4^ though the greatest problem was recognising and defining clinically significant vascular permeability. Acute dengue vasculopathy generally lasts for less than 48 hours, presenting physicians with an array of rapidly changing pathophysiological conditions.^5^ The patient must have multiple haematocrit observations, and a definitive diagnosis depends on the timing of these, making nonsense of the complaint that the WHO case definition requires too many ‘repeated clinical tests’.^1^

Microhaematocrit testing is critical to establishing a diagnosis of hypervolaemia owing to loss of fluid and to designing and managing fluid and colloid resuscitation.^6^ In most South-east Asian countries, microhaematocrit centrifuges are on treatment wards and used by ward personnel. However, in the Americas, haematocrit determinations require venepuncture and are performed in central laboratories, resulting in serious reporting delays. Therefore, much of the perceived problem in documenting dengue vascular permeability is owing to the organisation of hospital laboratory services.7,^8^

### The 2009 dengue case definition

**Figure 1:**
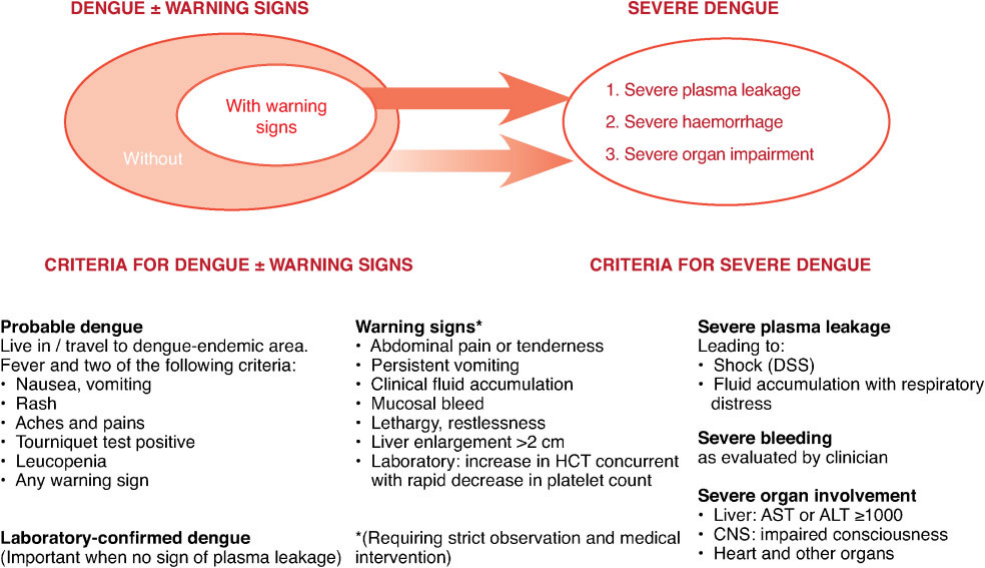
WHO 2009 suggested dengue case classification and levels of severity^9^

The 2009 revised WHO case definition (Fig. 1) has, however, created serious difficulties for the clinician and research scientist.^9^ This two-tiered definition consists of initial ‘warning signs’ and a catch-all category, ‘severe dengue’. Note the failure to supply specific quantitative diagnostic criteria and the reliance on individual clinical judgment. How is one to identify ‘clinical fluid accumulation’, ‘increase in haematocrit’ or ‘severe plasma leakage’? What is ‘narrow pulse pressure’ or ‘high haematocrit’?

For the clinician, the WHO recommends that all patients with any ‘warning sign’ should be hospitalised.^9^ Recent experience has demonstrated that compliance with this, particularly in medical communities with little prior experience of DHF, may lead to serious over-hospitalisation. This may then delay triage and recognition of patients requiring life-saving fluid resuscitation, resulting in a high case–fatality rate.^10^

While the new case definition may be useful for surveillance and reporting, there being no requirement for any laboratory studies, use of the 2009 WHO case definition to define a patient population using the term ‘severe dengue’ will destroy serious research on dengue pathogenesis. ‘Severe dengue’ substitutes a *mélange* of disease attributes, many end-stage, for the clinically unique and distinct dengue vascular permeability syndrome. Severe organ failure may result from blood loss. It is well documented that dengue fever in adults with peptic ulcer disease may be accompanied by severe focal gastro-intestinal (GI) bleeding, shock and death. Similarly, failure to detect and correct leaky capillaries may result in shock or compensated shock that shunts blood away from the GI tract, resulting in severe bleeding. If uncorrected, these two types of GI bleeding with different causal pathways (and completely different treatment) may result in severe shock, organ failure and encephalopathy. Furthermore, fluid accumulation with respiratory distress (owing to hypervolaemia and pulmonary oedema) is an end-stage outcome possibly resulting from mismanagement of fluid administration, i.e. too much intravenous fluid. To treat end-stage clinical observations as if they derive from a defined clinical syndrome is a serious mistake and has already led to research testing unfeasible pathophysiological hypotheses.^11^ Therefore, a case definition that discriminates between primary haemorrhage and vascular permeability is necessary for robust pathological research into the spectrum of dengue syndromes.

### (ii) The Correlation Between Dengue Vascular Permeability Syndrome (DHF/DSS) and a Secondary Dengue Infection is not Significant

In 1977, Rosen, an early critic of hospital-based observations of an association between a secondary-type antibody response to dengue infection and DHF/DSS, called for field-based studies to clarify the evidence.^12^ Since then, a number of retrospective sero-epidemiological studies have confirmed that severe dengue disease is associated with secondary dengue infections. This includes data gathered in the ‘favourable environment’ of Cuba, providing unequivocal evidence that individuals circulating dengue 1 antibodies were at risk of DHF during subsequent dengue 2 or dengue 3 infections.^13^–^15^

However, there are often problems with data collected in a hospital rather than a study setting, with around 10–30% of hospitalised DHF cases often classified as being caused by primary dengue infection.^16^ This could be owing to mis-labelling because of the retrospective nature of most of these data where DHF may be diagnosed without evidence of vascular permeability because observers assume that it is indicated by the presence of thrombocytopenia.^16^ Another explanation is serological misclassification as detection of primary and secondary antibody responses is often based on tests from a single sample of acute-phase serum.^16^

### (iii) DHF/DSS is Caused by Virulent Dengue Viruses

Significant efforts have been directed to finding genetically distinct viruses that cause severe or mild dengue disease. The four dengue virus strains (DENV1–4) vary in terms of pathogenicity and virulence, though the basis for these phenotypic differences is poorly understood. Pathogenicity describes the spectrum of disease syndromes associated with dengue infection. Island epidemics and human volunteer studies provide evidence that different strains within genotypes of dengue viruses vary greatly in intrinsic pathogenicity (i.e. in naive hosts).17,^18^ The ratio of DHF/DSS to total dengue infections can be measured and is referred to as virulence.

However, the relationship between second infections and dengue vasculopathy is complex. Not all sequential dengue infections result in DHF;^19^ this can be affected by host factors such as ethnicity20,^21^ or age22,^23^ and viral aspects, including timing^24^ or sequence^19^ of infection, along with heterotypic cross-protection following infection.25,^26^

In patients at risk of severe disease, the severity, or virulence, of dengue infections is regulated by the antibodies (whether actively or passively acquired). Homologous antibodies can provide complete protection, while heterotypic neutralising antibodies can down-regulate disease. It has also been observed that enhancing antibodies increase the infected cell mass and disease severity. However, it is not understood how this works at the molecular level.^27^

During the 1997 Santiago de Cuba outbreak caused by DENV2 infection in patients previously exposed to DENV1, the severity of disease increased month by month. The genetic sequences of viruses collected over the course of the epidemic and the serum neutralising antibodies were analysed.13,^28^ In this way, a single mutation in the non-structural genes of circulating DENV2 viruses might have contributed to viral survival or replication efficiency, thereby enhancing infection in the presence of antibodies.^28^ This process was described by the researchers as ‘increased viral fitness’, rather than virulence, and might increase the severity of the disease during an outbreak.^28^

### (iv) DHF is caused by Abnormal T-cell Responses

It has been proposed that, in dengue-infected individuals, abnormal and/or accelerated secondary T-cell responses leading to apoptosis contribute to increasing the severity of the immune elimination response.^29^–^33^ According to this hypothesis, T-cells from a first infection are inefficient at killing target cells infected with a second virus and would attack infected macrophages, leading to increased cytokine production. These cytokines would affect the vascular endothelium, ultimately causing thrombocytopenia and altered vascular permeability.^34^

However, in patients with DHF, circulating cytokine levels are similar in infants with primary dengue infections and children of any age with secondary dengue infection.^35^ DHF/DSS in infants is attributed to antibody-dependent enhancement of dengue infections.^36^–^38^ The ability of passively transferred dengue antibodies to enhance dengue viraemia has been demonstrated repeatedly in a monkey model.39,^40^ Higher levels of dengue plasma viraemia during early disease stages were associated with increased risk of DHF in children with secondary DENV3 infection during a hospital-based prospective study.^41^

Because primary dengue infections in infants result in authentic DHF, a secondary immune response is not required to produce this syndrome. T-cell researchers need to study infant DHF/DSS to find immunological mechanisms that unify primary- and secondary-infection DHF. Clearly, if T-cell responses contribute directly to vascular permeability, T-cells responding to a first infection must be as efficient as T-cell responses to heterologous infection. However, it might be that T-cells responding to primary infections renders inefficient their response to a secondary infection.^33^ Speculations that aberrant or abnormal T-cell responses cause DHF/DSS are unwarranted and unnecessary. It has long been noted that individuals with DHF are unusually healthy; surely their immune responses should be normal? Therefore, as two mechanisms cannot be responsible for the same pathology, aberrant or abnormal T-cell responses to dengue infection are not involved.

In the future, when time and effort are invested in studying the pathogenesis and immunology of infant DHF/DSS, it can be expected that a unified explanation will emerge. Meanwhile, it is important to remember that dengue infection in the presence of enhancing antibodies must produce an expanded infected-cell mass. T-cell responses, whether primary or secondary, should be proportional to this antigenic load.^42^

### (v) DHF/DSS Results from an Auto-immune Process

Currently, several mechanisms are proposed to explain auto-immune responses to viral infections, including molecular mimicry.^43^–^45^ Similarities have been observed between structural envelope and internal non-structural protein 1 (NS1) of dengue viruses and human proteins.^46^,47 Furthermore, antibodies to dengue NS1 proteins have been shown to react with plasminogen and integrin,^47^ platelets48,^49^ and endothelial cells.^49^

However, the hypothesis that this observed structural mimicry is involved in the development of severe disease is inconsistent with the epidemiology and evolution of DHF. For example, in infant DHF, antibodies to envelope or NS1 DENV proteins are unlikely to appear earlier than the fifth day after onset of fever. However, thrombocytopenia in these infants is regularly detected on the second or third day after onset of fever, while vascular permeability occurs around day five. Crucially, thrombocytopenia and vascular permeability ease just as quickly as they begin. It is impossible to understand how NS1 antibodies can produce transient thrombocytopenia and endothelial damage as a result of an antibody response that lasts for many years. If auto-immune responses are mediated by antibodies, why does this not produce chronic vascular permeability and thrombocytopenia?^50^

### (vi) DHF Results from Direct Infection of Endothelial Cells

If the hypothesis that dengue viruses replicate in endothelial cells is correct, it should be possible to observe viral antigens or virions within infected endothelial cells, as has been shown with other infections.^51^ As yet, however, no unequivocal evidence of DENV infection of endothelial cells *in vivo* has been shown. Initial findings apparently showing evidence of dengue in endothelial cells was revealed to be dengue antigens on the surface of cells labelled as endothelial cells, and secondary probes showed no evidence of infection within the cells.^52^

During secondary dengue infection, DENV replication has only been observed within human hepatocytes, monocytes and macrophages.^53^ Infection peaks after defervescence, with enhanced virus production resulting in a large cell mass. This attracts a massive T-cell response, leading to DSS.^54^

## Conclusions

Dengue infection and the associated spectrum of syndromes are associated with a number of controversies, some of which have been empirically resolved while others require further study. In particular, a clinically and physiologically applicable case classification that will allow robust pathological research into the different levels of disease severity is a major priority.

**Table:** Box 1 WHO 1997 case definitions for DF, DHF and DSS^55^

DF	**Probable**
	• An acute febrile illness with two or more of the following manifestations: headache, retro-orbital pain, myalgia, arthralgia, rash, haemorrhagic manifestations and leucopenia
	*and*
	• Supportive serology (a reciprocal haemagglutination-inhibition antibody titre ⩾1280, a comparable IgG enzyme-linked immunosorbent assay (ELISA, see chapter 4^55^) titre or a positive IgM antibody test on a late acute or convalescent-phase serum specimen)
	*or*
	• Occurrence at the same location and time as other DF cases
	**Confirmed**
	• A case confirmed by one of the following laboratory criteria:
	– Isolation of the dengue virus from serum/autopsy samples
	– At least a four-fold change in reciprocal IgG/IgM titres to one or more dengue virus antigens in paired samples
	– Demonstration of dengue virus antigen in autopsy tissue, serum or cerebrospinal fluid samples by immunohistochemistry, immunofluorescence or ELISA
	– Detection of dengue virus genomic sequences in autopsy tissue serum or cerebrospinal fluid samples by polymerase chain reaction (PCR)
	**Reportable**
	• Any probable or confirmed case should be reported
DHF	For a diagnosis of DHF, a case must meet all four of the following criteria:
	• Fever or history of fever lasting 2–7 days, occasionally biphasic
	• A haemorrhagic tendency shown by at least one of the following: a positive tourniquet test*; petechiae, ecchymoses or purpura; bleeding from the mucosa, gastro-intestinal tract, injection sites or other locations; haematemesis or melaena
	• Thrombocytopenia [⩽100,000 cells/mm^3^ (100×10^9^/L)]^†^
	• Evidence of plasma leakage due to increased vascular permeability shown by: an increase in the haematocrit ⩾20% above average for age, sex and population; a decrease in the haematocrit after intervention ⩾20% of baseline; signs of plasma leakage such as pleural effusion, ascites or hypoproteinaemia
DSS	For a case of DSS, all four criteria for DHF must be met, in addition to evidence of circulatory failure manifested by:
	• Rapid and weak pulse
	*and*
	• Narrow pulse pressure (<20 mmHg or 2·7 kPa)
	*or manifested by*
	• Hypotension for age
	*and*
	• Cold, clammy skin and restlessness

* The tourniquet test is performed by inflating a blood pressure cuff on the upper arm to a point midway between the systolic and diastolic pressures for 5 minutes. A test is considered positive when 20 or more petechiae per 2·5 cm^2^ (1 inch) are observed. The test may be negative or mildly positive during the phase of profound shock. It usually becomes positive, sometimes strongly positive, if the test is conducted after recovery from shock.

^†^ This number represents a direct count using a phase-contrast microscope (normal is 200,000–500,000/mm^3^). In practice, for outpatients, an approximate count from a peripheral blood smear is acceptable. In normal persons, 4–10 platelets per oil-immersion field (100×; the average of the readings from 10 oil-immersion fields is recommended) indicates an adequate platelet count. An average of 3 platelets per oil-immersion field is considered low (i.e. 100,000/mm^3^).
